# Genetic variants in vitamin D signaling pathways and risk of gestational diabetes mellitus

**DOI:** 10.18632/oncotarget.11984

**Published:** 2016-09-12

**Authors:** Aiwu Shi, Juan Wen, Guangquan Liu, Heng Liu, Ziyi Fu, Jing Zhou, Yao Zhu, Yaoqiu Liu, Xirong Guo, Jianguo Xu

**Affiliations:** ^1^ Department of Anaesthesiology, Jinling Hospital, School of Medicine, Nanjing University, Nanjing, China; ^2^ Department of MICU, Nanjing Maternity and Child Health Care Hospital Affiliated to Nanjing Medical University, Nanjing, China; ^3^ State Key Laboratory of Reproductive Medicine, Nanjing Maternity and Child Health Care Hospital Affiliated to Nanjing Medical University, Nanjing, China; ^4^ Nanjing Maternity and Child Health Care Institute, Nanjing Maternity and Child Health Care Hospital Affiliated to Nanjing Medical University, Nanjing, China; ^5^ Department of Children Health Care, Nanjing Maternity and Child Health Care Hospital Affiliated to Nanjing Medical University, Nanjing, China

**Keywords:** vitamin D, pathway, polymorphism, gestational diabetes mellitus, Pathology Section

## Abstract

Vitamin D (VD) deficiency during pregnancy has been repeatedly linked to an increased gestational diabetes mellitus (GDM) risk. We sought to determine the influences of genetic variants in vitamin D signaling pathways on the risk of GDM. In this study, we genotyped 15 single nucleotide polymorphisms (SNPs) within 8 representative genes (*CYP27A1*, *CYP27B1*, *CYP24A1*, *VDR*, *RXRA*, *RXRB*, *RXRG* and *GC*) of the vitamin D signaling pathways in a case-control study with 964 GDM cases and 1,021 controls using the Sequenom MassARRAY iPLEX platform. Logistic regression analyses in additive model showed that *GC* rs16847024 C>T, *RXRG* rs17429130 G>C and *RXRA* rs4917356 T>C were significantly associated with the increased risk of GDM (adjusted OR = 1.31, 95% CI = 1.10-1.58 for rs16847024; adjusted OR = 1.28, 95% CI = 1.04-1.57 for rs17429130; adjusted OR = 1.28, 95% CI = 1.06-1.54 for rs4917356). And GDM risk significantly increased with the increasing number of variant alleles of the three SNPs in a dose-dependent manner (*P* for trend < 0.001). Moreover, the combined effect of the three SNPs on GDM occurrence was more prominent in older women (age > 30). Further interactive analyses also detected a significantly multiplicative interaction between the combined variant alleles and age on GDM risk (*P* = 0.035). Together, these findings indicate that *GC* rs16847024, *RXRG* rs17429130 and *RXRA* rs4917356 were candidate susceptibility markers for GDM in Chinese females. Further validation studies with different ethnic background and biological function analyses were needed.

## INTRODUCTION

Gestational diabetes mellitus (GDM) is defined as glucose intolerance with onset or first recognition during pregnancy [1], which has become a significant health problem globally. It has been reported that GDM affects 1%-14% of all pregnancies, and that its incidence has been steadily rising [2]. GDM not just increase the risk of adverse pregnancy outcomes, but also has lots of long-term health impacts on both mothers and their offspring, including susceptibility to obesity, type 2 diabetes mellitus (T2DM) and metabolic syndrome in later life [3, 4]. Therefore, early intervention of the risk factors for GDM and early detection may be the key to improve the prognosis. Accumulating evidence has indicated that risk factors for GDM include advanced maternal age, pre-pregnancy overweight and obesity, history of subfertility or infertility and family history of diabetes [5, 6]. In addition, available data suggest that GDM have a familial tendency, and it recurs in at least 30% of women with a history of GDM [7, 8], supporting a genetic component in the etiology of GDM.

In the past decade, vitamin D (VD) deficiency and insufficiency during pregnancy has also been repeatedly linked to an increased GDM risk [9, 10]. Although the most widely accepted physiological role of VD is to maintain calcium and phosphate levels for bone formation [11], it is now clear that VD also has a range of non-calcitropic functions, such as stimulating insulin production and participating in the pathological processes of T2DM [12, 13]. In humans, a vast majority of VD is synthesized through photochemical conversion of 7-dehydrocholesterol to pre-vitamin D_3_ in the skin, and the latter is sequentially metabolized in the liver and kidneys [14]. The active metabolite of vitamin D, 1α,25(OH)_2_D_3_, is mainly produced by 2 hydroxylases: 25-hydroxylase (encoded by the gene *CYP27A1*) in the liver and 1α-hydroxylase (encoded by the gene *CYP27B1*) in the kidney. *CYP24A1* is strongly induced by the action of 1α,25(OH)_2_D_3_, plays an important role in the production of the less active VD metabolites. The Vitamin D receptor (VDR), a member of the steroid hormone receptor superfamily, functions as a transcriptional activator of numerous genes, which is essential for VD activity. The 1α,25(OH)2D3-VDR-dependent transcriptional activity is modulated through synergistic ligand-binding and dimerization with retinoic X receptor (RXR) [14]. Additionally, the VD-binding protein (group-specific component protein, GC), which serves to deliver VD to target tissues, is specifically responsible for VD endocytosis [15]. Recently, multiple loci in *CYP27A1*, *CYP27B1*, *CYP24A1*, *VDR*, *RXR* and *GC* genes were found to be associated with vitamin D levels [16]. It is reported that maternal serum VD levels increase up to twofold starting at 10-12 weeks' gestation and reaching a maximum in the third trimester, suggesting an important role for VD in obstetric well-being [17].

Numerous studies have suggested that VD plays important roles in β-cell function and impaired glucose tolerance in GDM [18, 19], therefore, we hypothesized that genetic variants in VD signaling pathways that influence VD levels could predispose to GDM. To test this hypothesis, we selected 15 single nucleotide polymorphisms (SNPs) within 8 representative genes (*CYP27A1*, *CYP27B1*, *CYP24A1*, *VDR*, *RXRA*, *RXRB*, *RXRG* and *GC*) encoding the core proteins involved in VD synthesis and catabolism, and performed a case-control study including 964 GDM cases and 1,021 controls to test the association between these polymorphisms and the risk of GDM.

## RESULTS

Selected characteristics of the 964 GDM cases and 1,021 controls are described in Table S1. As expected, there were similar distributions of age and pre-pregnancy body mass index (BMI) between the two groups (*P* = 0.094 and 0.685, respectively). However, there were more multiparous women among GDM cases than among controls (*P* < 0.001). And the rates of abnormal pregnancy history and family history of diabetes were also significant higher among GDM cases (*P* < 0.05 for all comparisons).

**Table 1:** Genotyping results in GDM cases and controls

SNP	Base chang^a^	Gene	Location	MAF^b^	Reported MAF^c^	*P* value		
						Dominant model	Recessive model	Additive model
rs2248137	C>G	CYP24A1	chr20q13.2	0.431	0.413	0.365	0.845	0.478
rs2259735	C>T	CYP24A1	chr20q13.2	0.309	0.330	0.675	0.479	0.996
rs4674343	A>G	CYP27A1	chr2q35	0.174	0.151	0.275	0.237	0.183
rs4646536	G>A	CYP27B1	chr12q14.1	0.357	0.364	0.389	0.662	0.671
rs4341603	G>T	VDR	chr12q13.11	0.408	0.403	0.140	0.433	0.153
rs7136534	C>T	VDR	chr12q13.11	0.369	0.408	0.106	0.500	0.137
rs739837	G>T	VDR	chr12q13.11	0.267	0.282	0.663	0.553	0.914
rs28465650	A>G	RXRA	chr9q34.2	0.162	0.180	0.536	0.996	0.599
rs34835001	T>C	RXRA	chr9q34.2	0.235	0.223	0.769	0.508	0.995
rs3818740	T>C	RXRA	chr9q34.2	0.217	0.199	0.708	0.165	0.838
**rs4917356**	T>C	RXRA	chr9q34.2	0.110	0.121	**0.036**	**0.020**	**0.011**
rs1805343	A>G	RXRA	chr9q34.2	0.341	0.335	0.333	0.580	0.654
rs166899	C>T	RXRG	chr1q23.3	0.212	0.180	0.079	0.326	0.067
**rs17429130**	G>C	RXRG	chr1q23.3	0.092	0.126	**0.044**	**0.035**	**0.017**
**rs16847024**	C>T	GC	chr4q13.3	0.120	0.141	**0.006**	0.074	**0.003**

Notes:

a Major > minor allele;

b MAF in 1021 controls;

c Reported MAF in Han Chinese from 1,000 genomes. Bold value denotes statistical significance.

Abbreviations: GDM, gestational diabetes mellitus; SNP, single nucleotide polymorphism; MAF, minor allele frequency.

Logistic regression analyses were used to examine the associations between the 15 studied SNPs and GDM susceptibility by using different genetic models. The observed genotype frequencies for the 15 SNPs among the controls were all in Hardy-Weinberg equilibrium (*P* > 0.05 for all SNPs). As shown in Tables 1-2, *GC* rs16847024 C>T, *RXRG* rs17429130 G>C and *RXRA* rs4917356 T>C were significantly associated with the increased risk of GDM using the additive model (adjusted OR = 1.31, 95% CI = 1.10-1.58 for rs16847024; adjusted OR = 1.28, 95% CI = 1.04-1.57 for rs17429130; adjusted OR = 1.28, 95% CI = 1.06-1.54 for rs4917356), but not others. We then used conditional logistic regression analyses to test the independence of the three significant SNPs. The effects of rs16847024, rs17429130 and rs4917356 on GDM occurrence remained in existence after being conditioned on the other two SNPs (Table S2).

**Table 2:** Association between 3 significant SNPs and GDM susceptibility

Genotype	GDM cases (*n* = 964) *N* (%)	Controls (*n* = 1021) *N* (%)	OR (95%CI)	*P*	OR (95%CI) a	*P*^a^
rs16847024						
CC	695 (72.4)	791 (77.8)	1.00		1.00	
CT	237 (24.7)	208 (20.5)	1.30 (1.05-1.60)	0.016	1.30 (1.04-1.61)	0.018
TT	28 (2.9)	18 (1.8)	1.77 (0.97-3.23)	0.062	1.83 (1.00-3.38)	0.051
Additive			1.31 (1.09-1.57)	0.003	1.31 (1.10-1.58)	0.003
rs17429130						
GG	760 (78.9)	838 (82.6)	1.00		1.00	
GC	182 (18.9)	166 (16.4)	1.21 (0.96-1.53)	0.109	1.20 (0.95-1.52)	0.128
CC	21 (2.2)	10 (1.0)	2.32 (1.08-4.95)	0.030	2.31 (1.07-5.00)	0.034
Additive			1.29 (1.05-1.57)	0.014	1.28 (1.04-1.57)	0.017
rs4917356						
TT	732 (76.5)	806 (79.4)	1.00		1.00	
CT	197 (20.6)	194 (19.1)	1.12 (0.90-1.40)	0.324	1.19 (0.95-1.49)	0.131
CC	28 (2.9)	15 (1.5)	2.06 (1.09-3.88)	0.026	2.18 (1.14-4.15)	0.018
Additive			1.21 (1.01-1.46)	0.042	1.28 (1.06-1.54)	0.011

Note:

a Logistic regression analyses adjusted for age, pre-pregnancy BMI, parity, abnormal pregnancy history and family history of diabetes

Abbreviations: GDM, gestational diabetes mellitus; SNP, single nucleotide polymorphism.

Then, we evaluated combined effects by adding up the number of variant alleles of the independent SNPs on GDM occurrence (rs16847024-T, rs17429130-C and rs4917356-C). The “0” allele means subjects with wide-type homozygotes of the three SNPs; “1-6” alleles means carrying one to six variant alleles of the three SNPs. As shown in Table 3, the risk of GDM was significantly increased with the increasing number of variant alleles of the three SNPs in a dose dependent manner (*P* for trend < 0.001). Subjects carrying “1-2” variant alleles had a 39% increase in GDM risk (95% CI = 1.16-1.67), compared with subjects with “0” allele. And subjects carrying “3-6” variant alleles had an even higher risk of GDM (adjusted OR = 1.98, 95% CI = 1.10-3.56), when compared with subjects with “0” allele (Table 3).

**Table 3:** Cumulative effects of variant alleles on GDM susceptibility

Variables	GDM cases (*n* = 964) *N* (%)	Controls (*n* = 1021) *N* (%)	OR (95%CI)	*P*	OR (95%CI) a	*P*^a^
Combined effects of rs16847024-T, rs17429130-C and rs4917356-C						
0	418 (43.77)	521 (51.84)	1.00		1.00	
1-2	507 (53.09)	464 (46.17)	1.36 (1.14-1.63)	0.001	1.39 (1.16-1.67)	< 0.001
3-6	30 (3.14)	20 (1.99)	1.87 (1.05-3.34)	0.035	1.98 (1.10-3.56)	0.023
Trend			*P*^b^ < 0.001		*P*^b^ < 0.001	

Note:

a Logistic regression analyses adjusted for age, pre-pregnancy BMI, parity, abnormal pregnancy history and family history of diabetes

b *P* value of Cochran-Armitage's trend test

Abbreviation: GDM, gestational diabetes mellitus; BMI, body mass index.

The combined effects of the three SNPs on GDM occurrence were also evaluated by stratifying on age, pre-pregnancy BMI, parity, abnormal pregnancy history and family history of diabetes (Table 4). Similar association strengths were shown between most subgroups (*P* > 0.05 for heterogeneity test). Interestingly, a stronger combined effect of the three SNPs on GDM occurrence was observed among older women (age > 30) (adjusted OR =1.68, 95% CI = 1.30-2.17) compared with that in younger women (age ≤ 30) (adjusted OR = 1.19, 95% CI = 0.95-1.49) (*P* = 0.047 for heterogeneity test). Further interactive analyses also detected a significantly multiplicative interaction between the combined variant alleles and age on GDM risk (*P* = 0.035) (Table 5). Crossover analysis suggested that the older women (age > 30) with “1-2” alleles (i.e., rs16847024-T, rs17429130-C and rs4917356-C) and “3-6” alleles were associated with significantly increased risk of GDM (adjusted OR = 2.02, 95% CI = 1.55-2.62, *P* < 0.001; adjusted OR = 5.75, 95% CI = 1.88-17.60, *P* = 0.002, respectively), as compared with the younger women (age ≤ 30) with “0” alleles (Table 5).

**Table 4:** Stratified analyses on the combined effects of the three SNPs with GDM susceptibility

Variables	0/1–2/3-6 allele (s)		OR (95%CI)	*P*^b^	OR (95%CI)^a^	*P*^b^
	GDM (*n* = 964) *N* (%)	Controls (*n* = 1021) *N* (%)				
Age, year						
≤ 30	221/246/14	314/289/16	1.18 (0.95-1.47)	0.052	1.19 (0.95-1.49)	0.047
> 30	197/261/16	207/175/4	1.64 (1.28-2.11)		1.68 (1.30-2.17)	
Pre-pregnancy BMI, kg/m^2^						
≤ 22	217/267/15	262/245/8	1.35 (1.08-1.70)	0.929	1.40 (1.11-1.78)	1.000
> 22	201/240/15	259/219/12	1.37 (1.09-1.73)		1.40 (1.11-1.78)	
Parity						
Nulliparae	358/434/27	481/436/20	1.34 (1.13-1.59)	0.281	1.34 (1.12-1.59)	0.497
Multiparae	60/73/3	40/28/0	1.86 (1.05-3.29)		1.68 (0.90-3.16)	
Abnormal pregnancy history						
No	362/450/27	499/448/19	1.39 (1.17-1.64)	0.884	1.39 (1.17-1.65)	0.903
Yes	56/57/3	22/16/1	1.32 (0.67-2.59)		1.46 (0.68-3.16)	
Family history of diabetes						
No	346/414/24	448/397/18	1.34 (1.12-1.60)	0.689	1.36 (1.14-1.64)	0.761
Yes	72/93/6	73/67/2	1.47 (0.97-2.23)		1.46 (0.96-2.22)	

Note:

a Logistic regression analyses adjusted for age, pre-pregnancy BMI, parity, abnormal pregnancy history and family history of diabetes (excluded the stratified factor in each stratum)

b *P* -value for the heterogeneity test. Abbreviations: GDM, gestational diabetes mellitus; SNP, single nucleotide polymorphism; BMI, body mass index.

**Table 5:** Interaction analyses on the combined effects of the three SNPs and age on GDM susceptibility

Alleles	Age	GDM cases (*n* = 964) *N* (%)	Controls (*n* = 1021) *N* (%)	OR (95%CI)	*P*
0	≤ 30	221 (23.1)	314 (31.2)	1.00	
1-2	≤ 30	246 (25.8)	289 (28.8)	1.21 (0.95-1.55)	0.122
3-6	≤ 30	14 (1.5)	16 (1.6)	1.27 (0.60-2.68)	0.532
0	> 30	197 (20.6)	207 (20.6)	1.24 (0.95-1.62)	0.115
1-2	> 30	261 (27.3)	175 (17.4)	2.02 (1.55-2.62)	< 0.001
3-6	> 30	16 (1.7)	4 (0.4)	5.75 (1.88-17.60)	0.002
Interaction				^a^ *P* = 0.035	

Note: Logistic regression analyses adjusted for pre-pregnancy BMI, parity, abnormal pregnancy history and family history of diabetes

a *P* -value for the heterogeneity test. Abbreviations: GDM, gestational diabetes mellitus; SNP, single nucleotide polymorphism.

## DISCUSSION

In this study, we investigated the association between genetic variants in vitamin D signaling pathways and risk of GDM in Southeast Han Chinese populations. We found that *GC* rs16847024 C>T, *RXRG* rs17429130 G>C and *RXRA* rs4917356 T>C were significantly associated with the increased risk of GDM. And the GDM risk significantly increased with the increasing number of variant alleles of the three SNPs in a dose-dependent manner.

To date, accumulating data has showed that VD deficiency has been related with numerous health outcomes, including heart disease, hypertension, autoimmune disease, infectious disease, cancer, type 1 diabetes, T2DM and GDM [20, 21]. Several studies have found that the genetic variants of the genes in VD signaling pathways were associated with the concentrations of VD [16]. In a case-control study conducted in the north of China demonstrated an association between GC variants and GDM, as well as a relation between a subset of loci in *CYP2R1*, *CYP24A1* and *VDR* and clinical parameters related to GDM [22]. Recently, a study performed on a population of pregnant Iranian women also showed a significant association between *VDR ApaI* and *TaqI* gene polymorphisms and the risk of GDM [23]. Furthermore, randomized clinical trial showed that VD supplementation could decrease the incidence of GDM [24], which might provide further evidence for the importance of the role of VD in the GDM occurrence.

Variants in *GC* have previously been reported in association with T2DM and quantitative traits connected with diabetes mellitus, including plasma glucose, insulin concentrations, and insulin resistance [25, 26]. The SNP rs16847024 (C>T) is located at the 5′flanking region of *GC*. According to the web-based SNP analysis tool (SNPinfo: http://snpinfo.niehs.nih.gov/snpinfo/snpfunc.htm), rs16847024 may be located at transcription factor binding site, which is likely to involve in gene regulation including promoter activation or repression depending on its interacted protein. In the present study, the allele-T conferred risk effect for the GDM occurrence at the rs16847024 locus. It has also been reported that rs16847024 was related to high-sensitivity C-reactive protein levels, which is one of important inflammatory factors [22]. RXR, retinoic X receptors, are transcription factors with important roles in development, reproduction, homeostasis, and cell differentiation. There are typically three copies of the gene, *RXRA* (*NR2B1*/*RXRα*), *RXRB* (*NR2B2*/*RXRβ*) and *RXRG* (*NR2B3*/*RXRγ*) [27]. We found rs17429130 in *RXRG* and rs4917356 in *RXRA* were associated with the GDM susceptibility. The SNP rs17429130 (G>C) is located at the 3′-untranslated region of *RXRG*. Interestingly, rs17429130 may be located at miRNA-binding site according to the above SNP analysis tool, which is likely to disrupt miRNA-target interaction and result in the deregulation of target gene expression. For rs4917356 (T>C), although it is located at the intron of *RXRA*, it is associated with the expression of RXRA according to the database of GTExPortal (http://www.gtexportal.org). These evidences for the three SNPs seems to be biologically plausible, but further functional analysis of the regions including the three SNPs, is needed. We also observed a significant interaction between the combined alleles and age on GDM risk. Advanced maternal age has been recognized as one of major causes of pregnancy complications, especially for GDM [5], and VD deficiency and insufficiency widely exists in the older pregnant women, which may help explain the interaction observed, although the underlying mechanisms are not fully elucidated.

GDM occurrence was the result of the combined effect of multiple risk factors and multiple genes, each of which could not be despised. Genome wide association (GWA) studies are proving adept at identifying common variants contributing to the inherited component of common diseases. Almost all such variants seem to have low or modest effect sizes [28]. In this study, the effects of the three SNPs (rs16847024, rs17429130 and rs4917356) were also modest. However it may be helpful to explain the “missing heritability” to some extent. Our study had a number of strengths. First of all, our GDM cases and controls came from a systematic screening of pregnancy complications in a large, population-based study conducted in Nanjing, and the two groups were well matched on age and pre-pregnancy BMI, which may have reduced potential selection bias. Moreover, the relatively large sample size in this study provided enough statistical power. In this study, the vitamin D level was not measured in all subjects, which was the major limitation. Multiple loci in VD signaling pathways (*CYP27A1*, *CYP27B1*, *CYP24A1*, *VDR*, *RXR* and *GC* genes) were found to be associated with vitamin D levels [16], so it is plausible that common variants in these genes that influence vitamin D level could predispose to GDM. Further studies involving the association of the three SNPs with vitamin D level are needed. And some reported risk factors for GDM, such as poor diet, low physical activity, polycystic ovarian syndrome [5, 6], were not considered for adjustment in this study. Therefore, the results should be treated with caution, and validations are warranted.

Taken together, our study suggested that *RXRA*, *RXRG*, and *GC* loci are candidate susceptibility regions that have some marker SNPs for GDM in Han Chinese. Further studies with functional assays conducted in diverse populations are needed to validate and extend our findings.

## MATERIALS AND METHODS

### Study subjects

The study was approved by the institutional review board of Nanjing Maternity and Child Health Care Institute (Nanjing, China), and the methods were carried out in accordance with the approved guidelines. The case-control study was conducted on the basis of a study population of more than 80,000 women who attending pregnancy complications screening between March 2012 and February 2015 in Nanjing Maternity and Child Health Care Hospital (Nanjing, China). The GDM cases and controls were randomly selected from the maternal screening population by using a computerized random number function. All participants were offered a glucose challenge test (GCT) between weeks 24 and 28 of gestation. The GDM cases were defined as the pregnant women with fasting glucose concentration ≥ 5.5 mmol/L or 2-h plasma glucose concentration ≥ 8.0 mmol/L [29]. Women diagnosed with diabetes before pregnancy were excluded from this study. The pregnant women without diabetes were included as controls. The selected controls were matched to the GDM cases on age and pre-pregnancy BMI, and declared no previous metabolic diseases. As a result, 964 GDM cases and 1021 controls consented to participate in the study. All cases and controls were unrelated ethnic Han Chinese. After written informed consent was obtained, each participant was scheduled for an interview by using a structured questionnaire to collect demographic information and potential risk factors, such as age, pre-pregnancy height and weight, parity, abnormal pregnancy history and family history of diabetes.

### SNPs selection and genotyping

Based on the data from UCSC database (GRCh37/hg19), common SNPs associated with core components (*CYP27A1*, *CYP27B1*, *CYP24A1*, *VDR*, *RXRA*, *RXRB*, *RXRG* and *GC*) of VD signaling pathways were included for initial analysis. The selected SNPs had minor allele frequency (MAF) larger than 0.05 in Chinese/Asians and were located at the 5′flanking regions, 5′-untranslated regions (5′-UTRs), coding regions, or 3′-UTRs according to NCBI dbSNP data (last search date: December 2015). We also included SNPs with biological significance or those that were associated with gene expression according to Regulome Database. If SNPs are in high linkage disequilibrium (r^2^ > 0.8), we would genotype only one SNP. As a result, 15 SNPs (rs2248137 and rs2259735 in *CYP24A1*; rs4674343 in *CYP27A1*; rs4646536 in *CYP27B1*; rs4341603, rs7136534 and rs739837 in *VDR*; rs28465650, rs34835001, rs3818740, rs4917356 and rs1805343 in *RXRA*; rs166899 and rs17429130 in *RXRG*; and rs16847024 in *GC*) were selected for genotyping (Table 1).

Genomic DNA was extracted from leukocyte pellets by traditional proteinase K digestion and followed by phenol-chloroform extraction and ethanol precipitation. All SNPs were genotyped using the Sequenom MassARRAY iPLEX platform (Sequenom Inc., CA). The information on primers is shown in Table S3. The genotyping assays was performed without knowing the subjects' case and control status. Two blank controls (water) in each 384-well plate were used for quality control and more than 10% samples were randomly selected to repeat, yielding a 100% concordance. The success rates of genotyping for these SNPs were all above 98.5%.

### Statistical analyses

Differences of selected characteristics and genotype frequencies of SNPs between the GDM cases and controls were calculated by the Student's *t*-test (for continuous variables) and χ^2^ test (for categorical variables). Both univariate and multivariate logistic regression analyses were used to estimate the associations between the genotypes and GDM risk by computing odds ratios (OR) and their 95% confidence intervals (CIs). The Cochran-Armitage test was used for trend analyses. The χ^2^-based Q test was used to assess the heterogeneity of associations among subgroups. All the statistical analyses were performed with SAS 9.1.3 software (SAS Institute, Cary, NC), and *P* < 0.05 in a two-sided test was considered statistically significant.

## SUPPLEMENTARY MATERIALS TABLES


