# Supplementary material for: Genetic variants in vitamin D signaling pathways and risk of gestational diabetes mellitus
Source: Oncotarget. 2016 Sep 12;7(42):67788–95. doi: 10.18632/oncotarget.11984 (PMC5356519; doi:10.18632/oncotarget.11984)
Supplement: Supplementary file 1 [file oncotarget-07-67788-s001.pdf]

Genetic variants in vitamin D signaling pathways and risk of gestational diabetes mellitus

Supplementary Material

Table S1 Demographic and selected variables in GDM cases and controls

Variables	GDM cases (n = 964) N (%)	Controls (n = 1021) N (%)	*P*
Age, year (mean ± SD)	30.6 ± 3.7	30.3 + 3.6	0.094
Pre-pregnancy BMI, kg/m^2 (mean ± SD)	22.1 ± 2.9	22.0 ± 2.8	0.685
Parity			< 0.001
Nulliparae	827 (85.8)	953 (93.3)	
Multiparae	137 (14.2)	68 (6.7)	
Abnormal pregnancy history			< 0.001
No	847 (87.9)	981 (96.1)	
Yes	117 (12.1)	40 (3.9)	
Family history of diabetes			0.023
No	791 (82.1)	876 (85.8)	
Yes	173 (17.9)	145 (14.2)	

Abbreviation: GDM, gestational diabetes mellitus; SD, standard deviation; BMI, body mass index.

Table S2 Conditional analyses on association between the three SNPs and GDM risk

	OR (95CI%) [a]	*P* [a]	OR (95CI%) [b]	*P* [b]	OR (95CI%) [c]	*P* [c]	OR (95CI%) [d]	*P* [d]
rs16847024	-	-	1.32 (1.10-1.59)	0.003	1.33 (1.10-1.59)	0.003	1.34 (1.11-1.61)	0.002
rs17429130	1.29 (1.05-1.58)	0.018	-	-	1.28 (1.05-1.57)	0.017	1.29 (1.05-1.59)	0.017
rs4917356	1.26 (1.04-1.52)	0.018	1.29 (1.07-1.57)	0.008	-	-	1.28 (1.06-1.56)	0.011

Note: Logistic regression analyses adjusted for age, pre-pregnancy BMI, parity, abnormal pregnancy history and family history of diabetes. [a] Logistic regression analyses conditioned on rs16847024; [b] Logistic regression analyses conditioned on rs17429130; [c] Logistic regression analyses conditioned on rs4917356; [d] Logistic regression analyses conditioned on the other two SNPs. Abbreviations: GDM, gestational diabetes mellitus; SNP, single nucleotide polymorphism.

Table S3 Information of primers for Sequenom MassARRAY iPLEX assays

SNP_ID	2nd-PCR Primer	1st-PCR Primer	Extend Primer
rs166899	ACGTTGGATGTCCAGCTCACTGTAGCTAAC	ACGTTGGATGACTGCAGCAATGCTTTGCTC	CCTTTTTTCCAGTGTCATCT
rs16847024	ACGTTGGATGCACTGGGTAAATTCTGTGAC	ACGTTGGATGAACCAGGAGTGGAACTCATC	GAGAGTCATAGGTAGATCATT
rs17429130	ACGTTGGATGACTTTCTGGAAGTTCTTGGG	ACGTTGGATGCATTTGTTTGCTTTCCAGGG	ATGGAAGTTCTTGGGAAAACTAAT
rs1805343	ACGTTGGATGAGCAAGAGCTTAGCGAACCT	ACGTTGGATGTCCAGTGCCAGGGCAGAACT	AAAAGGTCTGGTGAGGGC
rs2248137	ACGTTGGATGATGGTGCGCTTCGGCGTCT	ACGTTGGATGATCCTCCGCCGTGCCCGAA	TCGGCGTCTGGGCAG
rs2259735	ACGTTGGATGTTCTCTGGCTATTTCTTGGC	ACGTTGGATGAGAAATACTCCAGCTGCAAC	GGGTCTATTTCTTGGCATGATTCTC
rs28465650	ACGTTGGATGGGGAGTGTTTTACAGAATC	ACGTTGGATGAGGAGAGCATTCTCGGTGTG	GTTTTACAGAATCTCAGCA
rs34835001	ACGTTGGATGTTCCAGCTGCTGCCCGATTT	ACGTTGGATGAGAGCACGAGGGTATGACAG	GACACCGTCTGTGTGTG
rs3818740	ACGTTGGATGTTTTTCCCAGGCTTCCTGAG	ACGTTGGATGTCTCTAAGTCAAGCGAGTCC	GATGAGGCGAGGGCATCC
rs4341603	ACGTTGGATGGGCTGAATTTTTCAGGTCTC	ACGTTGGATGCCTTCAGGGAGATCCTTAAC	CAAGTTTTCAGGTCTCTCCTTTA
rs4646536	ACGTTGGATGGTTGGAAACAATGAGAAGGG	ACGTTGGATGCAGTCTAGGTTGCAAAGCAC	CCCTAGCCTCATCTTG
rs4674343	ACGTTGGATGCAGAACCAGAATATTGACCC	ACGTTGGATGCTGCCTGCTCCTGTGAACC	AGGTACCCAGATTTTTACATTATCC
rs4917356	ACGTTGGATGGGGTGATCTGCTTGTCCAGT	ACGTTGGATGTGAAACTCATCCCCACTGTC	GCTTGTCCAGTGGTTTC
rs7136534	ACGTTGGATGTTCTCAGGAACCAACTGCCG	ACGTTGGATGGAGAGTCCTGGTCCTAAGCA	CACTCCGCTTTCCCGC
rs739837	ACGTTGGATGCTGTTCCCTCAACATCAGTC	ACGTTGGATGAGGGCCTTGCCCAGAGATG	GGGAAGTCAGCAGCCACTTA
